# Assessing Pain in Children: A Narrative Review of Distraction‐Based Interventions and Outcome Measurement Tools

**DOI:** 10.1002/pne2.70048

**Published:** 2026-09-26

**Authors:** Meryem Akay Kuzey

**Affiliations:** ^1^ College of Nursing and Health Sciences Murray State University Murray Kentucky USA

**Keywords:** AI‐based interactive games, distraction‐based interventions, non‐pharmacological methods, pediatric pain assessment

## Abstract

Procedural pain and fear are common during pediatric immunizations and other needle‐related procedures. This narrative review examined distraction‐based, non‐pharmacological interventions and the outcome measures used to assess pain and fear in children aged 6–12 years, with particular attention to AI‐based interactive games and the Buzzy/Buzzle Bee cold‐vibration device. A structured search of multiple databases identified 30 peer‐reviewed studies published between 2015 and 2024. Studies were synthesized narratively according to intervention type, pain/fear outcomes, and measurement approach. Technology‐based distraction, particularly virtual reality (VR), was associated with reduced pain, fear, or anxiety across multiple studies. Cold‐vibration devices also reduced procedural pain and distress, while low‐cost distraction methods provided practical alternatives. Outcome measures included self‐report pain scales, fear scales, behavioral measures, proxy ratings, and physiological indicators. The findings support distraction‐based approaches while highlighting substantial variation in outcome measurement and the need for consistent, developmentally appropriate assessment tools. Although these interventions show promise, the studies vary widely in how pain and fear are measured. The studies included used a variety of outcome measures, including self‐report pain scales, fear scales, behavioral assessment tools, proxy ratings, and physiological indicators, making direct comparisons across studies more difficult. These inconsistencies highlight the need for more standardized and developmentally appropriate outcome measures. By summarizing current evidence and identifying gaps in assessment practices, this review underscores the importance of using reliable, child‐centered tools to evaluate distraction‐based interventions. Integrating these evidence‐based distraction strategies into routine pediatric care may help reduce procedural trauma, improve children's experiences, and support more child‐centered care.

## 1 Introduction

Procedural pain is one of the most common sources of distress for children in healthcare settings. Routine interventions such as venipuncture and immunizations often provoke fear, anxiety, and behavioral resistance, which can prolong procedures and create lasting negative associations with healthcare [1, 2, 3]. When pain is not managed well in childhood, children may develop needle phobia, avoid medical care, or experience heightened anxiety during future encounters. Supporting children through these procedures is important not only for comfort in the moment but also for building trust between the child, family, and healthcare team [4].

Pharmacological methods, including topical anesthetics, can help reduce discomfort but may be limited by availability, cost, time, or side effects [5]. As a result, many clinicians have turned to non‐pharmacological, distraction‐based methods that target the psychological and sensory components of pain [6, 7]. These strategies work by shifting the child's focus away from the procedure, a concept supported by the gate control theory of pain, which suggests that competing sensory input can reduce the transmission of pain signals [8].

Recent studies have drawn attention to technology‐based distraction and cold‐vibration devices as non‐pharmacological options for pediatric procedural pain. AI‐based interactive games were selected as a focal technology because they represent an emerging, interactive approach to distraction, although direct evidence in pediatric pain is limited in the included literature. Accordingly, related VR studies were also considered because they provide evidence on immersive, technology‐based distraction, but VR was treated as a related intervention rather than as direct evidence of AI efficacy [9, 10]. The Buzzy/Buzzle Bee device was selected as a second focal approach because it represents a distinct cold‐vibration method and has been evaluated in multiple pediatric procedural studies, including immunization‐related research. Although commercially available, its inclusion was based on its pediatric procedural evidence and relevance to immunization‐related pain management rather than its commercial status [11, 12]. Simple and inexpensive tools—such as bubble blowing, ball squeezing, and kaleidoscope viewing—were also considered to provide context for established distraction approaches [7, 13].

Although many distraction‐based interventions show positive results, there is substantial variation in how outcomes such as pain, fear, and anxiety are measured. Studies employ a wide range of tools, including the Wong‐Baker FACES Pain Rating Scale, the Children's Fear Scale, behavioral observations, and physiological indicators such as heart rate or cortisol levels [2, 14]. These tools differ in reliability, sensitivity, and developmental suitability. The lack of standardized assessment approaches limits comparability across studies and makes it difficult to identify which interventions are most effective for specific age groups or procedures.

Growing evidence supports the use of distraction methods, including cold application and interactive technologies, in reducing children's procedural pain. Studies evaluating these techniques consistently report meaningful reductions in pain and distress during routine procedures [5, 12, 13, 14, 15]. The desired outcomes (O) for this population include lower levels of pain and fear—both important for preventing long‐term negative associations with healthcare and minimizing psychological effects linked to needle‐related procedures [16].

The purpose of this narrative review is to address two related questions: (1) what evidence supports distraction‐based, non‐pharmacological interventions—particularly AI‐based interactive games, related VR technology, and the Buzzy/Buzzle Bee device—for reducing procedural pain and fear; and (2) what outcome measures are used to assess pain and fear among children aged 6–12 years. The review was designed to synthesize evidence across these two domains and identify implications for developmentally appropriate, child‐centered pediatric care.

In this review, child‐centered strategies refer to developmentally appropriate interventions that actively involve children in managing pain and fear while considering their cognitive, emotional, and individual needs during medical procedures. For school‐aged children, a child‐centered approach includes offering understandable choices when feasible, using age‐appropriate distraction, and selecting pain and fear measures that allow children to communicate their experiences. The 6‐ to 12‐year age range was selected because children in this developmental period can often self‐report pain and fear while actively engaging with interactive distraction strategies such as games and VR [2, 9, 10].

By synthesizing current evidence, this review aims to highlight areas of consistency and inconsistency, identify gaps in outcome measurement, and offer guidance on integrating evidence‐based distraction strategies into pediatric clinical practice to improve the procedural experience for children and their families.

## 2 Narrative Review Methods

### 2.1 PICOT Question

Managing pain and anxiety during immunizations is an important part of supporting children's overall healthcare experience and reducing procedural distress [13, 16]. This review examines two non‐pharmacological interventions—AI‐based interactive games and the Buzzle Bee device—used to lessen pain and fear in pediatric patients during immunization. The target population (P) includes children aged 6–12 years, an age group that responds well to sensory, cognitive, and engaging distraction strategies. These approaches offer a way to manage pain and anxiety without the risks or limitations associated with pharmacological treatments.

The guiding PICOT question for this review is: *In pediatric patients aged 6–12 years undergoing immunizations (P), how do AI‐based interactive games and the Buzzle Bee device (I) compare with each other (C) in reducing pain and fear (O) during and immediately after the immunization procedure (T)?*

The PICOT question defines the primary comparison between AI‐based interactive games and the Buzzle Bee device, while the broader narrative synthesis also considers related technology‐based and established distraction interventions identified in the literature.

### 2.2 Search Criteria

A structured literature search was conducted for this narrative review on non‐pharmacological methods for managing pain, fear, and anxiety during pediatric procedures, with particular attention to immunization and needle‐related care. The search covered PubMed, MEDLINE, ScienceDirect, CINAHL, the Cochrane Library, Web of Science, Google Scholar, the National Library of Medicine (NIH), and the American Academy of Pediatrics (AAP). These sources were selected to provide broad coverage of pediatric, nursing, medical, and interdisciplinary literature relevant to the review question.

The inclusion criteria centered on peer‐reviewed studies published primarily between 2019 and 2024, with three earlier studies from 2015 and 2016 retained because of their foundational relevance to pediatric procedural pain. Eligible studies evaluated non‐pharmacological distraction or cold‐vibration interventions in pediatric populations and reported pain, fear, anxiety, behavioral, or physiological outcomes relevant to the review aims. Randomized controlled trials, systematic reviews, meta‐analyses, and other empirical studies were eligible. Studies with broader age ranges were retained when the study population included the target school‐aged population and the findings were relevant to the review question.

Exclusion criteria included studies focused exclusively on pharmacological pain management, studies that did not include the target school‐aged population or relevant pediatric findings, non‐full‐text articles, books, and studies unrelated to non‐pharmacological procedural pain or distress. Studies combining distraction with topical anesthetics or other pharmacological interventions were excluded when the independent contribution of the distraction intervention could not be identified; studies were retained when the distraction effect could be separately evaluated. Systematic reviews and meta‐analyses were included as secondary evidence sources; this review did not use an umbrella‐review or review‐of‐reviews design.

Search terms included pediatric immunization pain, pediatric procedural pain, pain management, non‐pharmacological interventions, AI‐based games for children, virtual reality pain management, distraction techniques, and Buzzy/Buzzle Bee. The search process included removal of duplicate records, title and abstract screening, and full‐text evaluation. A total of 30 studies were included for narrative synthesis. Because the included evidence varied in intervention, procedure, population, and measurement approach, findings were not statistically pooled.

The search criteria were used to maintain alignment with the review aims and PICOT question. Detailed search results and screening information are summarized in Table A1 in the Appendix A.

A detailed overview of the search process is provided in the Appendix A. Table A1 summarizes the search terms, limits, and screening results across all databases, showing how studies were identified, screened, excluded, and selected. Table A2 presents the level of evidence for each included study, outlining the design, sample, setting, primary findings, and measured outcomes. Together, these tables provide a clear and structured representation of how evidence was gathered, evaluated, and selected for inclusion in this review.

In addition to applying inclusion and exclusion criteria, the included studies were appraised according to study design, level of evidence, methodological clarity, sample characteristics, and appropriateness of outcome measurement tools. The narrative synthesis was organized deductively around the two review aims and three prespecified domains: (1) type of distraction intervention, (2) effects on pain, fear, or anxiety, and (3) outcome measurement approaches. Findings were compared across studies and grouped by intervention type and measurement domain rather than statistically pooled. Randomized controlled trials, systematic reviews, and meta‐analyses were prioritized when drawing conclusions, while lower‐level evidence was used to provide contextual information. Table A2 presents the study‐level evidence appraisal.

The review therefore used a structured narrative, deductive synthesis rather than a formal meta‐analysis. For each included study, information was extracted on the intervention, population and setting, procedure, pain/fear outcomes, measurement tools, major findings, and feasibility. The extracted evidence was then compared within intervention categories and across outcome‐measurement categories. This approach allowed the review to address both the effectiveness of distraction‐based interventions and the variation in tools used to measure pain and fear.

### 2.3 Evidence Summary: Non‐Pharmacological Interventions for Pediatric Procedural Pain and Anxiety

Technology‐based distraction was supported most strongly by studies of VR, while direct evidence on AI‐based interactive games was more limited. The included literature contained limited direct evidence on AI‐based interactive games, whereas Tas et al. [10] and Lluesma‐Vidal et al. [9] synthesized evidence on immersive VR. Across these studies, technology‐based distraction was associated with reductions in pain, fear, or anxiety. VR findings are therefore interpreted as evidence for technology‐based distraction and as contextual evidence for the broader category, not as direct evidence that AI‐based games and VR are equivalent interventions.

Several studies further support VR's benefits across different care settings. Wong and Choi [17] showed that VR reduced distress during venipuncture in school‐aged children. Dumoulin et al. [18] confirmed its usefulness in emergency departments, where rapid interventions are often needed. A large meta‐analysis by Eijlers et al. [19] found consistent reductions in pain and anxiety across pediatric populations, although issues such as cost and equipment availability were noted. Johnson [20] also highlighted VR's potential when adequate technological resources are available. Additional comparisons by Sarı et al. [21] showed that VR was more effective than kaleidoscopes in reducing pain, while kaleidoscopes offered comparable anxiety relief. Yaz et al. [22] found that VR and puppetry both reduced pain, with VR having a stronger effect on anxiety. Gerçeker et al. [23] further noted that VR lowered pain, fear, and negative emotional responses during phlebotomy.

Cold‐vibration devices, including Buzzy and Buzzle Bee, provided another evidence‐supported non‐pharmacological approach. Ahmed et al. [4] found that Buzzy combined with visual distraction reduced pain during phlebotomy, while Şahiner et al. [11] reported reductions in pain and anxiety during immunization. Semerci et al. [12] also reported reductions in procedural pain and fear. At the same time, evidence was not uniformly positive across procedures and age groups; Yılmaz et al. [24] reported limited effectiveness during catheterization in older children. These findings suggest that cold‐vibration devices should be considered in relation to the child's age, procedure, and clinical context.

Low‐cost distraction tools remain valuable in many pediatric settings. Techniques such as soap bubble blowing, ball squeezing, kaleidoscope viewing, and auditory stimuli have demonstrated clear benefits in reducing pain and fear [7]. Karakaya and Gözen [25] found that distraction significantly reduced pain levels in school‐aged children undergoing venipuncture. Binay et al. [13] showed that soap bubble blowing helped decrease fear and anxiety during venipuncture, while Girgin and Göl [6] highlighted ball squeezing as useful for mild procedural pain. Cheraghi et al. [14] found that visual distractions were slightly more effective than auditory stimuli during burn dressing changes. Sarı et al. [21] noted that kaleidoscopes can be a practical alternative when VR is not available. Arıkan and Işık Esenay [26] supported the benefits of distraction during venous blood sampling, showing meaningful reductions in pain.

Broader reviews reinforce these findings. Suleiman‐Martos et al. [27] and Guillari et al. [28] showed that VR and cold‐vibration devices consistently outperform standard care in reducing procedural pain and anxiety. Cozzi et al. [15] offered practical recommendations for integrating distraction methods into routine practice. These results align with Gerçeker et al. [23], who demonstrated that VR remains effective even when paired with simple tools such as stress balls, though accessibility challenges may persist in resource‐limited environments.

A detailed synthesis of the included studies is provided in Table A2 in the Appendix A. The table summarizes the purpose, design, sample, setting, major variables, measurement tools, data analysis, level of evidence, and feasibility of each intervention. Most studies were randomized controlled trials or high‐level systematic reviews and meta‐analyses, with additional cohort and quasi‐experimental designs contributing contextual insight. Across these studies, non‐pharmacological strategies—such as VR, game‐based interventions, cold‐vibration devices, and simple distraction methods like soap bubble blowing and ball squeezing—were consistently associated with reductions in pain, fear, and anxiety and were generally rated as feasible and low‐risk in routine pediatric practice. At the same time, Table A2 highlights important limitations, including small samples, single‐site designs, and variability in procedures and outcome measures, underscoring the need for larger, multi‐site trials and greater standardization in pediatric pain assessment.

### 2.4 Outcome Measures Used to Assess Pain and Fear

The included studies used several categories of outcome measures to assess pain, fear, and anxiety. Self‐report pain measures included the Wong‐Baker FACES Pain Rating Scale, Oucher Pain Scale, and Visual Analog Scale (VAS). The Children's Fear Scale was commonly used to assess procedural fear. Behavioral measures included FLACC and observational assessments, while some studies used parent, caregiver, or nurse ratings as proxy assessments. Physiological indicators included heart rate, oxygen saturation, and salivary cortisol. For children aged 6–12 years, self‐report measures are particularly relevant because many school‐aged children can communicate pain and fear directly; however, observational and proxy measures may provide complementary information when communication, developmental differences, or procedural circumstances limit self‐report. Across the included literature, no single outcome‐measurement approach was used consistently.

Although the studies evaluated similar outcomes, the measurement tools varied substantially in scale type, reporter, timing, and construct. Some studies measured pain only, whereas others assessed fear, anxiety, behavioral distress, or physiological responses. This heterogeneity limits direct comparison of intervention effects and supports the use of clearly defined, developmentally appropriate outcome measures in future pediatric research.

### 2.5 Appraisal of Evidence

The evidence base for non‐pharmacological interventions is supported by multiple randomized controlled trials and systematic reviews, although its strength varies by intervention, procedure, population, and study design.

Much of the evidence is based on randomized controlled trials (RCTs) and systematic reviews, which strengthen confidence in the findings across different clinical settings.

Virtual reality (VR) and AI‐based interactive games consistently demonstrate effectiveness in reducing pain and anxiety by engaging children's attention. However, their use depends on access to equipment, staff training, and technological resources, which may limit feasibility in some clinical environments.

Cold‐vibration devices, such as the Buzzy and Buzzle Bee, are also well supported in literature. These devices are practical, cost‐effective, and particularly beneficial for younger children undergoing minimally invasive procedures. Their effectiveness, however, may decrease in older children or during more invasive procedures, highlighting the need to match the intervention to the clinical context.

Simple distraction methods—including soap bubble blowing, kaleidoscopes, stress balls, and auditory or visual stimuli—remain valuable options. These approaches are inexpensive, easy to implement, and adaptable across a wide range of settings, although they may be less effective for procedures involving higher levels of pain or distress.

Despite the overall strength of the evidence, several gaps remain. Direct comparisons between AI‐based games, cold‐vibration devices, and other distraction methods are limited, making it difficult to determine the most effective intervention for specific situations. In addition, most studies focus on short‐term outcomes, with limited attention to long‐term psychological effects. The frequent use of single‐site studies and well‐resourced settings also raises concerns about generalizability and feasibility in more diverse clinical environments.

### 2.6 Synthesis of Evidence

Taken together, the 30 included studies support non‐pharmacological distraction approaches for reducing procedural pain, fear, or anxiety in children, while also demonstrating important variation by intervention and clinical context. The strongest and most consistent evidence in the included literature concerned VR and cold‐vibration approaches, whereas direct evidence for AI‐based interactive games was limited. Simple distraction methods also showed practical benefits, particularly when low‐cost approaches were needed.

The review retains its comparative focus on AI‐based interactive games and the Buzzy/Buzzle Bee device, but the available evidence does not support treating AI‐based games and VR as interchangeable interventions. VR provided the larger body of technology‐based evidence and was consistently associated with reductions in pain and anxiety across several studies and reviews. Direct evidence for AI‐based interactive games was more limited. Cold‐vibration devices also demonstrated reductions in immediate procedural pain and distress, with some variation by age and procedure. Thus, the synthesis supports both approaches as promising, but it does not establish that one is superior to the other.

To provide a clear overview of how these interventions compare across the literature, Table A3 summarizes which non‐pharmacological methods were evaluated in each study. This table highlights the breadth of evidence supporting VR and cold‐vibration devices, while also showing the growing use of interactive games and traditional distraction strategies.

It also emphasizes the relatively limited number of studies specifically examining AI‐based game interventions, reinforcing the need for further research in this area.

A more detailed breakdown of each study is presented in Table A4, which outlines the design, sample characteristics, setting, key findings, and main outcomes. Most included studies are based on high‐level research designs, such as randomized controlled trials, clinical trials, systematic reviews, and meta‐analyses, which strengthens the overall reliability of the findings. Table A4 also reveals common patterns across studies, including consistent reductions in pain and fear, the importance of age‐appropriate interventions, and the potential benefits of combining techniques (e.g., Buzzy with visual distraction).

Overall, the tables show consistent patterns across studies, with most interventions demonstrating reductions in pain and fear. VR and AI‐based interactive approaches tend to show stronger effects on engagement and anxiety, while cold‐vibration devices are particularly effective for immediate pain relief. Simple distraction methods also provide meaningful benefits, especially in resource‐limited settings.

Together, Tables A3 and A4 help synthesize the evidence by clearly mapping the types of interventions across the literature and providing structured details about each study's contributions. These tables show that while VR and cold‐vibration devices have the strongest and most consistent support, all distraction‐based interventions offer meaningful benefits when tailored to the child's developmental level, the clinical setting, and the type of procedure.

These findings further support the comparative roles of AI‐based interactive games and the Buzzle Bee device within the PICOT framework guiding this review.

### 2.7 Contribution of This Review

Although many studies have explored distraction‐based interventions for reducing pain and fear in children, few reviews have examined these techniques alongside the outcome measurement tools used to assess their effectiveness. Existing research often focuses on a single intervention, a narrow patient age group, or one type of clinical procedure, making it difficult for clinicians to compare strategies or select the most appropriate method for routine immunizations. In addition, the literature shows wide variation in how pain, fear, and anxiety are measured—using different self‐report scales, behavioral tools, and physiological indicators. These inconsistencies limit the ability to compare findings across studies and create uncertainty about which interventions are most effective for school‐aged children.

This review contributes to the field by bringing together three components: (1) a focused examination of AI‐based interactive games and the Buzzy/Buzzle Bee device; (2) a broader synthesis of distraction‐based interventions used during pediatric needle‐related procedures; and (3) an examination of the outcome measurement tools used to assess pain and fear. By explicitly addressing the second aim, the review identifies how different measurement approaches may influence interpretation and comparability of intervention findings.

By integrating these elements, the review provides a more complete picture of how non‐pharmacological interventions perform and how their effects are measured in children aged 6–12 years. The inclusion of two comprehensive tables (Tables A3 and A4) offers a structured map of the interventions used across studies and highlights the level of evidence supporting each method. This organization allows clinicians and researchers to easily identify intervention patterns, gaps in study design, and areas where evidence is strong or limited.

The review also highlights a key gap in current practice: the need for more consistent, developmentally appropriate, and validated tools to assess pain and fear during pediatric immunizations. Without standardization, it becomes difficult to determine which distractions truly make the greatest difference in clinical practice. By drawing attention to these limitations, this review supports future research aimed at improving both intervention delivery and the measurement of outcomes.

Overall, the contribution of this work lies in its focused comparison of emerging technology and cold‐vibration approaches, its synthesis of established distraction methods, and its examination of pain and fear measurement. A child‐centered approach in this context means matching the intervention and assessment method to the child's developmental level, communication ability, preferences when feasible, and procedural needs. The findings therefore support not only distraction as an intervention strategy but also developmentally appropriate assessment as an essential part of evaluating pediatric pain and fear.

The variability in pain and fear assessment methods also has important implications for interpreting effectiveness. Differences in self‐report, behavioral, and physiological measures may lead to inconsistent findings across studies, making it difficult to determine the true impact of specific interventions. In some cases, the choice of measurement tool may influence reported outcomes, potentially overestimating or underestimating effectiveness. This variability limits comparability and highlights the need for more standardized and developmentally appropriate assessment approaches.

Another important limitation in the current literature is the focus on short‐term outcomes. Most studies assess pain and anxiety only during or immediately after procedures, with limited evidence on long‐term psychological effects or sustained benefits of these interventions. This gap makes it difficult to understand their lasting impact on children's healthcare experiences.

## 3 Implications for Practice

Incorporating non‐pharmacological interventions into pediatric procedural pain management can significantly improve children's comfort and overall experiences. AI‐based games and virtual reality (VR) should be considered in settings with the technological capacity to support them, particularly for school‐aged children who can actively engage with immersive environments [10, 29]. Adequate staff training and reliable access to equipment are essential for successful implementation [20].

Cold‐vibration devices such as the Buzzy and Buzzle Bee offer practical options for younger children during minimally invasive procedures. Their use should be tailored to the child's age, anxiety level, and the complexity of the procedure to achieve the best outcomes [4, 11].

In resource‐limited settings, low‐cost distraction methods—soap bubble blowing, kaleidoscopes, ball squeezing, and auditory cues—remain accessible and effective [13, 21]. These approaches may be combined with other strategies to increase effectiveness, especially for older children or procedures that are more complex [25].

Future research should directly compare the relative effectiveness of VR, cold‐vibration devices, and simple distraction techniques across various age groups and clinical contexts. Longitudinal studies are also needed to understand the longer‐term psychological effects of these interventions. Additionally, research on feasibility and cost‐effectiveness in low‐resource environments will be important to support equitable access to high‐quality pediatric pain management options [27, 28].

To enhance clinical applicability, pain and fear should be assessed with developmentally appropriate tools that allow the child's experience to be represented directly whenever possible. For school‐aged children, self‐report tools such as the Wong‐Baker FACES Pain Rating Scale can be useful when the child can reliably communicate pain. Behavioral observation and proxy ratings can complement self‐report when communication or procedural circumstances limit direct reporting. Using clearly defined and consistent measures across studies and clinical settings may improve comparability and strengthen evaluation of distraction‐based interventions.

## 4 Conclusion

Children's experiences with routine procedures such as immunizations can shape their long‐term attitudes toward healthcare. This review highlights the value of distraction‐based, non‐pharmacological interventions—including AI‐based interactive games, virtual reality, cold‐vibration devices, and simple, low‐cost tools—in reducing pain, fear, and anxiety during these moments. Across the literature, these strategies consistently improve comfort and help children cope more effectively, especially when the intervention is matched to the child's developmental stage and the demands of the procedure.

Despite the progress reflected in recent studies, important gaps remain. The evidence is heterogeneous in intervention type, procedure, study design, and outcome measurement. The findings specifically show that pain and fear were assessed with multiple self‐report, behavioral, proxy, and physiological measures, limiting direct comparisons across studies. Future research should use clearly defined, developmentally appropriate, and more consistent assessment approaches and should directly compare intervention types when feasible.

Overall, the evidence suggests that integrating distraction‐based methods into routine practice is both achievable and beneficial. By adopting these approaches, healthcare providers can help reduce procedural trauma, support a more positive care experience, and promote long‐term trust in the healthcare system among pediatric patients and their families.

## 5 Limitations

Although this review provides a comprehensive synthesis of distraction‐based interventions for pediatric procedural pain, several limitations should be acknowledged. The included studies varied widely in design, sample size, setting, and outcome measurement tools, which makes direct comparison across studies challenging. Many studies were conducted in single institutions or well‐resourced environments, limiting generalizability to clinics with fewer staff, limited technology, or higher patient volumes. In addition, only a small number of studies directly compared AI‐based modalities, virtual reality, cold‐vibration devices, and simple distraction methods, leaving questions about their relative effectiveness. The reliance on self‐report scales in younger children—who may have difficulty communicating pain—further complicates interpretation. Finally, because the literature on AI‐based games is still emerging, the evidence base remains smaller than that for VR or cold‐vibration devices. These limitations highlight the need for larger, multi‐site trials with consistent assessment tools and more attention to feasibility in real‐world clinical environments.

In addition, potential publication bias should also be considered, as studies with positive findings are more likely to be published. Language restrictions may also have influenced the selection of studies, as only articles available in English were included, which may limit the comprehensiveness of the review.

## 6 Strengths of the Review

This narrative review offers several strengths. A structured, multi‐database search strategy ensured broad coverage of current evidence from nursing, pediatric, and interdisciplinary sources. The review included high‐quality studies such as randomized controlled trials, systematic reviews, and meta‐analyses, strengthening confidence in the findings. By synthesizing both technologically advanced interventions and low‐cost alternatives, the review provides practical guidance for diverse practice settings. Additionally, the inclusion of Tables A1, A2, A3, A4 allows clear visualization of the evidence selection process, intervention types, study characteristics, and levels of evidence. Together, these strengths provide a solid foundation for understanding current practice and identifying areas where further research is needed.

## Funding

The author has nothing to report.

## Conflicts of Interest

The author declares no conflicts of interest.

## Data Availability

The author has nothing to report.

## Appendix A

**TABLE A1:** Database search criteria and screening results.

Database	Vocabulary	Meaningful limits	Records				Eligibility		Number of included studies
			Total number	After duplicates removed	Number of screened	Number of exclude	Number of eligible full‐text articles	Number of excluded eligible full‐text articles	
PubMed	Pain management in pediatric	6–12 age	50 articles	45	10	35	4	3	1
Web of Science	Pain and Fear	Nursing	1162 journals	1162	8	1154	5	3	2
PubMed	Pediatric pain	Nursing + 6–12 age	28 084 articles	2743	5	2738	4	1	3
Web of Science	Reducing pain and fear	In children + nursing	477 articles	61	6	55	4	3	1
Web of Science	Children undergoing phlebotomy	Pain + nursing	134	35	3	32	3	2	1
Web of Science	Pain anxiety	In pediatric	2569	21	2	19	2	1	1
Web of Science	Venipuncture	Pediatric + non‐pharmacologic	328	57	11	46	3	1	2
Medline	Pain management	Pediatric	562	38	8	30	4	1	3
ScienceDirect	Non‐pharmacological interventions	6–12 age	1276	546	15	511	5	3	2
Google Scholar	Virtual reality	Pain management	171	28	13	15	3	2	1
NIH			469	151	11	140	4	2	2
Cochrane Library	Immunizations	Pediatric + fear	36 532	1758	10	5	5	1	4
AAP	AI‐based games	Pediatric pain management	15	5	5	1	4	2	2
CINAHL	Pain distraction techniques	Pediatric + nursing	754	389	8	381	12	7	5

*Note:* This table summarizes the search terms, meaningful limits, total records retrieved, number of records screened, excluded articles, and the final number of eligible studies included in the review across all databases.

**TABLE A2:** Evidence appraisal of studies on distraction‐based non‐pharmacological interventions for pediatric procedural pain.

Citation: Author, date of publication and title	Purpose of study	Conceptual framework	Design/Method	Sample/Setting	Major variables studied and their definitions	Measurement of major variables	Data analysis	Findings	Level of evidence (LOE)	Strengths/Weaknesses	Feasibility	Conclusion	Recommendation
Alemdar and Aktaş [5]	Evaluate the efficacy of Buzzy, Jet Lidocaine, bubble‐blowing, and aromatherapy in reducing pain, stress, and fear in pediatric patients during venipuncture	It is based on existing knowledge regarding non‐pharmacological interventions	Randomized Control Trial (RCT)	Pediatric patients aged 5–10 years undergoing venipuncture in a maternity and children's hospital	Independent Variables: Non‐pharmacological interventions (Buzzy, Jet Lidocaine, etc.); Dependent Variables: Pain, fear levels	Oucher Pain Scale, Children's Fear Scale (CFS), salivary cortisol analysis was conducted to evaluate stress levels	RM‐ANOVA	Significant effect of Buzzy and bubble‐blowing on reducing pain and fear during venipuncture	Level I	Strength: Randomized design, multiple non‐pharmacological interventions, objective measures of stress (salivary cortisol) and subjective measures of pain and fear. Weakness: Small sample size, conducted in a single hospital	High feasibility for clinical implementation. The interventions are cost‐effective, easy to administer, and non‐invasive, making them practical for use in pediatric settings	Supports using non‐pharmacological interventions during venipuncture	Non‐pharmacological methods should be considered as first‐line interventions in pediatric settings
Carr et al. [1]	Investigate age and gender differences in pediatric pain and fear during medical procedures	Integration of psychological, developmental, and physiological aspects	Cohort Study	Pediatric patients, ages 3–12, in hospital settings	Independent Variables: Age, Gender; Dependent Variables: Pain and fear ratings	Face Pain Scale (Three‐faces), Thermometer visual analog scale (VAS), and Physiological recordings (Marshall 92 Automatic Oscillometric Sphygmomanometer, Patient information sheet)	Multivariate analyses of variance (MANOVAs), *t*‐test	Younger (aged 3–7) and older (aged 8–12) children reported similar amounts of pain and fear. Girls reported more pain than boys	Level II	Strength: The study included both self‐reported and physiological measures. Weakness: Potential for bias in self‐reported pain ratings in younger children (aged 3–7).	Feasible for large‐scale hospital implementation	Age and gender should be considered when assessing children's pain and fear	Pain and fear are distinct constructs in children and should be assessed separately, particularly when considering gender and age differences
Cohen et al. [2]	Review evidence‐based assessments for pediatric pain management	Evidence‐Based Practice (EBP) Model	Systematic Review (examining 17 different pain assessment tools)	Various settings, focusing on pediatric pain assessments (reviews a broad range of settings and populations related to pediatric pain, including infants, children, and adolescents, with various pain measures.)	Independent Variables: Pain management strategies; Dependent Variables: Pain intensity	Various validated pain scales (e.g., FLACC, VAS, Faces Pain Scale)	Systematic Review	The effectiveness of various pain assessment tools and mention cognitive‐behavioral techniques as useful strategies	Level I	Strength: Comprehensive review Weakness: Heterogeneity across studies	High feasibility for integration into practice	Cognitive‐behavioral techniques are recommended for procedural pain	Prioritize EBP interventions that are non‐pharmacological
Ahmed et al. [4]	Evaluate the effect of Buzzy and watching cartoons on venipuncture pain among children undergoing phlebotomy	Integration of psychological, developmental, and physiological aspects	Quasi‐experimental	The sample consisted of 150 school‐age children undergoing phlebotomy at pediatric emergency units	Independent Variables: Buzzy, watching Cartoons; Dependent Variables: Pain levels	Wong‐Baker Faces Pain Rating Scale	Kruskal Wallis and post hoc tests	Both Buzzy and cartoons significantly reduced pain, with Buzzy being more effective	Level II	Strength: Controlled comparison; Weakness: Limited to specific interventions	High feasibility (The interventions are feasible for clinical practice)	Both Buzzy and cartoons were effective in reducing pain during venipuncture	Recommend integrating Buzzy and cartoons into pediatric care and calls for further studies
Girgin and Göl [6]	Evaluate the effectiveness of distraction techniques such as balloon inflation, ball squeezing, and coughing in reducing procedural pain and fear in children undergoing venipuncture	Integration of cognitive‐behavioral distraction techniques and physiological modulation of pain perception to reduce venipuncture pain and fear in children	Randomized Control Trial (RCT)	Pediatric patients, ages 7–12, undergoing venipuncture	Independent Variables: distraction techniques used (ball squeezing, balloon inflation, coughing), Dependent Variables: Pain and fear levels	Wong‐Baker Faces Pain Scale, Children's Fear Scale (CFS)	*t*‐tests, ANOVA	Distraction techniques (balloon inflation, ball squeezing, and coughing) significantly reduced both pain and fear during venipuncture compared to the control group	Level I	Strength: Strong evidence for distraction methods; Weakness: Narrow age range	The feasibility of using simple distraction techniques like balloon inflation and ball squeezing in pediatric settings due to their minimal cost and ease of implementation	Distraction should be incorporated as a standard intervention as they are effective in reducing pain and fear	Cognitive distraction can enhance clinical outcomes
Oluc and Arslan [7]	Evaluate the effects of two different distraction methods (soap bubble blowing and ball squeezing) in reducing pain and fear during phlebotom	Integration of psychological, developmental, and physiological aspects	Randomized Control Trial (RCT)	The sample consisted of 111 children aged 6–12 years undergoing phlebotomy in an emergency department	Independent Variables: Two distraction techniques (soap bubble blowing and ball squeezing) Dependent Variables: Pain, fear levels	Wong‐Baker FACES Pain Rating Scale and Children's Fear Scale	The data were analyzed using repeated measures analysis of variance (ANOVA) and multiple comparisons	Both soap bubble blowing, and ball squeezing significantly reduced pain and fear compared to the control group. However, soap bubble blowing was more effective in reducing fear than ball squeezing	Level I	Strength: Strong Comparative design Weakness: The limitation of not being a double‐blind study and having a short follow‐up period.	High feasibility. The article suggests that both distraction methods are practical and easy to apply in clinical settings, with high feasibility for use in busy hospital units	Both methods are viable in clinical practice. The method of soap bubble blowing was found to be a more effective method in reducing fear	Distraction techniques are recommended for routine use
Schechter et al. [3]	The study's purpose is to review evidence‐based practices to reduce pain and fear during pediatric immunizations	Evidence‐Based Practice (EBP) Model	Systematic Review	Pediatric patients during immunizations	Independent Variables: Pain reduction strategies; Dependent Variables: Pain and stress levels	Various pain scales (e.g., VAS, Wong‐Baker)	Systematic review	Behavioral interventions reduce pain	Level I	Strength: Comprehensive review Weakness: Lack of specific focus on immunizations	Feasible for broader pediatric practice	Behavioral strategies, such as distraction and parental demeanor, are effective in reducing pain during pediatric immunizations	Broader application of behavioral interventions, including distraction techniques and parental involvement, to reduce pain and distress during immunizations
Tas et al. [10]	Investigate the effectiveness of VR as an intervention tool to reduce pain and anxiety in pediatric patients during medical procedures	Applying empirical methods to assess VR's effectiveness	Systematic Review and Meta‐Analysis	The study includes pediatric patients (children and adolescents) undergoing various medical procedures. It extended the earlier review's search to 2020, leading to the inclusion of 26 articles	Independent Variables: Virtual reality interventions; Dependent Variables: Pain, anxiety	Visual Anxiety scales, VAS for pain	Meta‐analysis with random‐effects model	Virtual reality significantly reduces pain and anxiety	Level I	Strength: Meta‐analysis and VR is effective across various procedures Weakness: Limited focus on specific procedures like venipuncture	High feasibility in specialized settings (VR is highly feasible for use in clinical pediatric settings)	VR is an effective tool for managing pain and anxiety in pediatric patients during medical procedures	Recommend expanding use of virtual reality in pediatric care
Yaz et al. [22]	Study the effects of vibrating cold application and puppet use on pain and fear during phlebotomy in children	Developmentally appropriate and multidimensional interventions in pediatric pain management, making it a well‐rounded and practical approach for addressing the distress caused by phlebotomy in young children	Randomized Control Trial (RCT)	The sample was 105 children aged 3–6 undergoing phlebotomy in a pediatric unit	Independent Variables: Vibrating cold application, puppet use; Dependent Variables: Pain and fear levels	Wong‐Baker Faces Pain Rating Scale and Children's Fear Scale	Kruskal‐Wallis test, ANOVA, and post hoc tests	The study found that both Bee Buzzy and puppets were effective in reducing pain and fear, although Bee Buzzy was more effective for pain	Level I	Strength: Multiple interventions Weakness: Limited to small sample size	Feasible in most pediatric settings	The article concludes that both methods were effective, with Bee Buzzy more effective for pain reduction	The study recommends the use of Bee Buzzy and puppets in pediatric venipuncture for pain and fear reduction
Yılmaz et al. [8]	Assess the effect of Buzzy on pain and satisfaction in blood donors during phlebotomy	This study integrates gate control theory and distraction theory to evaluate the effectiveness of the Buzzy device in reducing pain and increasing satisfaction during phlebotomy through the application of vibration and cold stimuli	Randomized Control Trial (RCT)	The study included 90 healthy adult males voluntarily donating blood at a university blood center	Independent Variables: Buzzy; Dependent Variables: Pain levels, satisfaction	VAS for pain, satisfaction scale to assess satisfaction levels	Kruskal‐Wallis test and post hoc analysis for comparing pain and satisfaction scores across groups	The study found a statistically significant reduction in pain and an increase in satisfaction in the Buzzy group compared to the control groups	Level I	Strength: Strong control of variables Weakness: Adult focus, limited pediatric data	Feasible for implementation in phlebotomy	Buzzy is effective in reducing pain and increases satisfaction during phlebotomy	Recommend extending research to different populations, particularly children
Young [30]	Reviews pediatric procedural pain and provides evidence‐based strategies for its management	N/A	Review Article	The article addresses pain in pediatric settings, focusing on routine and emergency medical procedures, such as immunizations and venipunctures	Independent Variables: Pain management strategies; Dependent Variables: Pain and anxiety	Visual Analog Scale (VAS), Faces scales, and behavioral pain scales	Descriptive analysis	The article provides a detailed review of the current understanding of pediatric pain and offers evidence‐based recommendations for management	Level VI	Strength: Broad overview Weakness: No specific intervention focus	Feasible across pediatric settings	Pain management should be a priority for pediatric care	Recommend further studies on long‐term impacts of procedural pain
Ahmed et al. [4] Effect of Buzzy and watching cartoons on venipuncture pain among children undergoing phlebotomy.	To evaluate the effectiveness of Buzzy and watching cartoons in reducing pain and anxiety in children during venipuncture	Pain	Randomized Controlled Trial	Pediatric patients aged 4–12 years undergoing venipuncture in an outpatient pediatric clinic in Egypt	Independent Variables: Buzzy and cartoon interventions; Dependent Variables: Pain and anxiety levels	Wong‐Baker FACES Pain Scale, parental assessments, and observer ratings	RM‐ANOVA	Significant reduction in pain and anxiety for both interventions, with the combined use of Buzzy and cartoons being the most effective	Level II	Strengths: Randomized design, use of multi‐method assessments for pain and anxiety; Weaknesses: Single‐center study, lack of long‐term follow‐up	High feasibility for implementation in clinical settings due to cost‐effectiveness and ease of administration	Supports the combined use of Buzzy and visual distractions (cartoons) for reducing pain and anxiety during pediatric venipuncture	Recommend integrating Buzzy with visual distractions in pediatric outpatient clinics to improve pain management
Binay et al. [13] Comparison of the effectiveness of two different methods of decreasing pain during phlebotomy in children: A randomized controlled trial	To compare the effectiveness of external cold and vibration versus blowing soap bubbles in reducing pain in children during phlebotomy	Gate Control Theory of Pain	Randomized Controlled Trial	96 children aged 3–6 years, university hospital pediatric phlebotomy department in Turkey	Independent Variables: External cold and vibration (Buzzy device), blowing soap bubbles; Dependent Variable: Pain levels during phlebotomy	Wong‐Baker FACES Pain Rating Scale, parental and nurse assessments	Kruskal‐Wallis test	Pain scores were significantly lower in both intervention groups compared to the control group; no significant difference between the two intervention methods	Level II	Strengths: Randomized, multi‐assessor; Weaknesses: Limited to one center, narrow age range	Highly feasible for use in clinical pediatric settings as both methods are low‐cost and quick to administer	Both external cold and vibration and blowing soap bubbles are effective non‐pharmacological methods for reducing procedural pain in children	Recommend integrating either method in clinical settings to improve pediatric pain management during needle procedures
Cheraghi et al. [14]. A Comparison of the Effect of Visual and Auditory Distractions on Physiological Indicators and Pain of Burn Dressing Change Among 6–12‐Year‐Old Children: A Clinical Trial Study	To compare visual and auditory distraction methods in reducing pain and stabilizing physiological indicators during burn dressing changes in children	Gate Control Theory of Pain	Single‐blind clinical trial	120 children (6–12 years) in a burn unit, Iran	IVs: Visual and auditory distraction; DV: Pain intensity, physiological indicators (heart rate, oxygen saturation)	Oucher pain scale, pulse oximetry for heart rate, and oxygen saturation	SPSS‐16, ANOVA, Bonferroni post hoc test	Both distractions significantly reduced pain, with visual distraction showing stronger effects on pain reduction and physiological stability	Level II	Strength: Rigorous control with a substantial sample; Weakness: Limited to one hospital and cultural context	High; non‐invasive, low‐cost distraction methods suitable for clinical settings	Visual and auditory distraction are effective for reducing pain and improving physiological stability during painful procedures in pediatric burn care	Implement visual distraction for optimal pain relief and physiological stability in pediatric burn dressings
Cozzi et al. [15] A narrative review with practical advice on how to decrease pain and distress during venepuncture and peripheral intravenous cannulation	To provide a comprehensive review of interventions for minimizing pain and distress during venipuncture and intravenous cannulation in children	Cognitive Behavioral Theory	Narrative Review	Studies involving pediatric venipuncture and intravenous cannulation	Independent: Intervention techniques; Dependent: Pain and distress reduction	Observational Scale of Behavioral Distress‐Revised, various pain scales	Narrative synthesis	Highlighted the effectiveness of distraction, physical comfort, and parental involvement in reducing procedural pain and distress	Level V	Strengths: Comprehensive review covering psychological, pharmacological, and physical techniques; Weaknesses: Not a systematic review, so lacks rigorous evidence grading	High feasibility due to low‐cost, practical interventions applicable in various settings	Emphasizes the importance of preparation, parental support, and distraction techniques in pediatric procedural pain management	Recommend implementing distraction and comfort techniques routinely in pediatric settings to reduce pain and distress
Dumoulin et al. [18] A randomized controlled trial of the use of virtual reality for needle‐related procedures in children and adolescents in the emergency department	Evaluate the efficacy of virtual reality (VR) as a distraction tool for reducing pain and distress during needle‐related procedures	Pain	Randomized Controlled Trial	94 children and adolescents (ages 6–17 years) undergoing needle‐related procedures in an emergency department	Independent Variables: Use of VR; Dependent Variables: Pain and distress levels	Wong‐Baker FACES Pain Scale, Children's Anxiety and Pain Scale (CAPS)	Paired *t*‐tests and ANOVA	VR significantly reduced pain and distress during needle‐related procedures compared to standard care	Level II	Strengths: Randomized design, diverse age range, use of validated scales; Weaknesses: Single‐center study, potential variability in VR engagement	Highly feasible for emergency departments with appropriate technological infrastructure	Supports VR as an effective distraction tool for managing needle‐related procedural pain and distress in pediatric emergency settings	Recommend implementing VR in emergency departments with appropriate training for staff to enhance pediatric care
Eijlers et al. [19] Meta‐analysis of Virtual Reality in Pediatrics: Effects on Pain and Anxiety	To assess the effectiveness of virtual reality (VR) on reducing pain and anxiety in pediatric patients during medical procedures	Distraction Theory	Systematic Review and Meta‐Analysis	Pediatric patients across multiple studies and various settings	Independent Variables: Use of VR; Dependent Variables: Pain and anxiety during medical procedures	Pain measured with Visual Analog Scale (VAS) and Faces Scale; anxiety assessed with standardized anxiety scales	Random‐effects model for pooled effect sizes	VR significantly reduced both pain (SMD = −1.30) and anxiety (SMD = −1.32) in pediatric patients during procedures	Level I	Strengths: Large sample, high effect sizes; Weaknesses: Heterogeneity in study methods and settings	High feasibility in clinical settings with VR resources	VR is an effective non‐pharmacological tool for reducing pain and anxiety in pediatric procedures	Recommend VR use in pediatric settings for pain and anxiety management, with further research to explore additional applications
Erdogan and Aytekin Ozdemir [31]. The Effect of Three Different Methods on Venipuncture Pain and Anxiety in Children: Distraction Cards, Virtual Reality, and Buzzy	To evaluate the effectiveness of distraction methods (Distraction Cards, VR, and Buzzy) in reducing pain and anxiety during venipuncture in children aged 7–12 years	Gate Control Theory	Randomized Controlled Trial	142 children aged 7–12 years undergoing venipuncture in a university hospital	Independent Variables: Non‐pharmacological interventions (Distraction Cards, VR, Buzzy) Dependent Variables: Pain and anxiety levels during venipuncture	Visual Analog Scale (VAS), Wong‐Baker FACES Pain Rating Scale, and Children's Fear Scale (CFS)	ANOVA and post hoc tests	Buzzy group reported the lowest pain and anxiety scores, followed by VR and Distraction Cards groups, indicating effectiveness in pain and anxiety reduction	Level I	Strengths: Randomized design, multiple raters, well‐defined scales for pain and anxiety Weaknesses: Single location study, non‐blinded design	High feasibility due to simplicity of interventions and ease of application	Non‐pharmacological interventions like Buzzy, VR, and Distraction Cards can be effective in reducing pain and anxiety during pediatric procedures	Recommend using Buzzy as a primary method for reducing procedural pain and anxiety in children, with VR and Distraction Cards as alternative options
Gerçeker et al. [23] The effects of virtual reality and stress ball distraction on procedure‐related emotional appearance, pain, fear, and anxiety during phlebotomy in children: A randomized controlled study	To evaluate the effects of virtual reality (VR) and stress ball distraction on pain, fear, anxiety, and emotional behavior in children undergoing phlebotomy		Randomized Controlled	150 children aged 7–12 years in a pediatric phlebotomy clinic in Turkey	Independent Variables: VR and stress ball interventions; Dependent Variables: Pain, fear, anxiety, and emotional behaviors during phlebotomy	Children's Emotional Manifestation Scale, Wong‐Baker FACES Pain Scale, Children's Fear Scale (CFS), and Child Anxiety Scale	ANOVA and post hoc tests	VR significantly reduced pain, fear, and anxiety compared to both the control and stress ball groups. VR also reduced negative emotional behaviors post‐procedure. Le	Level I	Strengths: Randomized design, use of validated scales; Weaknesses: Single setting, no long‐term follow‐up data	High feasibility in pediatric settings with access to VR technology	VR is effective in reducing pain, fear, anxiety, and negative emotional behaviors in children, showing potential for clinical application in pediatric phlebotomy	Recommend VR as a non‐pharmacological intervention in pediatric phlebotomy to manage pain, fear, and anxiety effectively
Guillari et al. [28] Non‐pharmacological interventions to reduce procedural needle pain in children (6–12 years):	To evaluate the effectiveness of non‐pharmacological interventions in reducing procedural pain in children aged 6–12 years	Pain	Systematic Review	15 studies including 1356 pediatric patients across various healthcare settings	Independent Variables: Various non‐pharmacological interventions (e.g., VR, cold‐vibration devices); Dependent Variables: Pain and anxiety levels	Wong‐Baker FACES Pain Scale, Children's Anxiety and Pain Scale (CAPS)	Meta‐analysis with subgroup comparisons	VR and cold‐vibration devices were the most effective interventions for reducing procedural pain and anxiety, particularly in school‐aged children	Level I	Strengths: Comprehensive review, inclusion of multiple high‐quality RCTs; Weaknesses: Variability in study designs and intervention protocols	High feasibility in clinical settings with appropriate resources and training	Non‐pharmacological interventions, particularly VR and cold‐vibration devices, are effective tools for reducing pain and anxiety in pediatric populations	Recommend integrating VR and cold‐vibration devices into pediatric procedural pain management strategies to enhance patient comfort
Johnson [20] Virtual reality for pain management in children	To review the application of virtual reality (VR) as a pain management intervention for children undergoing medical procedures	Pain	Narrative Review	Not applicable	Major Variable: Efficacy of VR in reducing procedural pain and anxiety in pediatric populations	Literature analysis from existing studies on VR applications in pediatric settings	Descriptive analysis	VR is a promising tool for reducing pain and anxiety in children, with significant potential for integration into various pediatric healthcare settings	Level V	Strengths: Comprehensive review of existing evidence; Weaknesses: Lacks empirical data and randomized trials	Feasible for implementation in pediatric units with adequate technological resources and trained staff	Supports the adoption of VR as a non‐pharmacological intervention to manage pain and anxiety in pediatric patients	Recommend expanding the use of VR in pediatric care and conducting further empirical studies to establish long‐term efficacy
Kara et al. [29] Interactive Games and Pediatric Pain Management.	To evaluate the effectiveness of interactive games in reducing procedural pain and anxiety in pediatric patients	Cognitive Behavioral Theory	Randomized Controlled Trial	120 pediatric patients aged 6–12 years undergoing venipuncture in a hospital setting	Independent Variable: Interactive games; Dependent Variables: Pain and anxiety levels	Wong‐Baker FACES Pain Scale, Visual Analog Scale (VAS)	Independent *t*‐tests and ANOVA	Interactive games significantly reduced pain and anxiety compared to standard care, with greater engagement observed among younger children	Level II	Strengths: Randomized design, use of validated pain scales; Weaknesses: Limited to a single center and specific procedural context	Feasible for pediatric settings with access to interactive game tools and trained staff	Supports the integration of interactive games into procedural pain management for pediatric patients	Recommend implementing interactive games as a non‐pharmacological intervention to enhance pediatric procedural experiences
Karakaya and Gözen [25] The effect of distraction on pain level felt by school‐age children during venipuncture procedure: A randomized controlled trial	To examine the impact of distraction techniques on procedural pain experienced by school‐aged children during venipuncture	Pain	Randomized Controlled Trial	80 school‐aged children (6–12 years old) in a hospital outpatient setting	Independent Variable: Distraction techniques; Dependent Variable: Pain levels during venipuncture	Wong‐Baker FACES Pain Scale	Chi‐square test and ANOVA	Distraction techniques significantly reduced pain levels compared to the control group, with higher efficacy in younger children	Level II	Strengths: Robust randomized design, use of validated tools; Weaknesses: Single‐center study, limited generalizability	Easily implementable in pediatric outpatient clinics with minimal training required for staff	Distraction techniques are effective and should be included as a standard practice in pediatric venipuncture procedures	Recommend training staff on distraction methods to enhance procedural outcomes for children
Le May et al. [32] Decreasing pain and fear in medical procedures with a pediatric population	To evaluate the effectiveness of VR in reducing pain and fear during pediatric medical procedures	Gate Control Theory	Pilot Randomized Within‐Subject Trial	30 pediatric patients aged 6–12 years in a hospital setting	Independent Variable: Virtual Reality (VR); Dependent Variables: Pain and fear levels during procedures	Wong‐Baker FACES Pain Scale and self‐reported fear ratings	Paired *t*‐tests and ANOVA	VR significantly reduced pain and fear compared to the control group receiving standard care	Level II	Strengths: Within‐subject design eliminates between‐group variability; Weaknesses: Small sample size limits generalizability	Feasible for settings equipped with VR technology and trained staff	VR is an effective tool for reducing pain and fear in pediatric procedures and should be considered in clinical settings	Recommend further large‐scale studies to validate findings and explore broader implementation
Lluesma‐Vidal et al. [9] Effect of Virtual Reality on Pediatric Pain and Fear During Procedures Involving Needles	To assess the effectiveness of virtual reality (VR) as a distraction tool in reducing pain and fear in pediatric patients during needle‐related procedures	Gate Control Theory of Pain	Systematic Review and Meta‐Analysis	21 studies reviewed, including 10 for meta‐analysis with a total sample of pediatric patients across various healthcare settings	Independent Variable: VR intervention; Dependent Variables: Pain and fear levels during needle procedures	Visual Analog Scale (VAS) for pain, fear scales as reported in individual studies	Meta‐analysis of pooled data with statistical adjustments	VR was effective in significantly reducing pain (mean reduction of 2.37) and fear (mean reduction of 1.26) in children undergoing needle procedures	Level I	Strengths: High statistical power, pooled data from multiple RCTs; Weaknesses: Variability in study quality, challenges with blinding participants	High feasibility in clinical settings where VR equipment is accessible	VR is an effective non‐pharmacological tool for reducing pain and fear in pediatric procedures involving needles	Recommend integrating VR as a standard option for pain and anxiety reduction during pediatric needle‐related procedures where feasible
[33] Virtual reality vs. Buzzy: Efficacy in pain and anxiety management during pediatric venipuncture	To compare the effectiveness of VR and Buzzy device as distraction methods during venipuncture in managing pain and anxiety among pediatric patients	Based on non‐pharmacological interventions and pain management literature	Systematic Review & Meta‐Analysis	21 studies with a total sample of 2504 pediatric patients (age 2–18) from various healthcare settings	Independent Variable: Type of distraction method (VR vs. Buzzy) Dependent Variables: Pain and anxiety levels	Pain measured by Visual Analog Scale (VAS) and Wong‐Baker FACES Pain Rating Scale; Anxiety measured by Children's Fear Scale (CFS)	Meta‐analysis	Both VR and Buzzy significantly reduced pain and anxiety compared to standard care; no significant difference in efficacy between VR and Buzzy	Level I	Strengths: Comprehensive analysis of 21 studies, robust statistical analysis. Weaknesses: High heterogeneity among studies	High feasibility for clinical implementation in pediatric settings, cost‐effective options for pain management	Supports VR and Buzzy as effective non‐pharmacological interventions for managing pain and anxiety during venipuncture in children	Further research recommended on cost–benefit, adverse effects, and satisfaction levels
Moadad et al. [34] Distraction Using the BUZZY for Children During an IV Insertion	To examine the effect of the BUZZY device on pain perception during IV insertions in children	Pain management through sensory distraction	Randomized Controlled Trial	48 children aged 4–12 years, hospital setting	Independent Variable: Use of BUZZY device (vibration and cold application); Dependent Variable: Pain scores during IV insertion	Children, parents, and nurses rated pain using standardized pain scales	*t*‐test comparing pain scores between BUZZY and control groups	BUZZY group showed significantly lower pain scores compared to control, indicating effective pain reduction	Level II	Strengths: Randomized design, multi‐rater assessments; Weaknesses: Small sample size, single‐site study	High feasibility for clinical application due to the simplicity of the BUZZY device	BUZZY is effective for reducing pain perception during IV insertions in children	Recommend integrating BUZZY as a standard intervention for pediatric IV procedures to minimize procedural pain
Oluc and Arslan [7] The effect of two different methods on reducing the pain and fear during phlebotomy to children: A randomized controlled trial	Evaluate the effectiveness of two distraction methods (soap bubble blowing and ball squeezing) in reducing pain and fear in children undergoing phlebotom	Based on non‐pharmacological distraction methods in pediatric pain management	Randomized Controlled	111 children aged 6–12 years in an emergency department in Turkey	Independent Variables: Soap bubble blowing, ball squeezing; Dependent Variables: Pain and fear levels during phlebotomy	Wong‐Baker FACES Pain Scale, Children's Fear Scale	ANOVA	Both soap bubble blowing and ball squeezing significantly reduced pain and fear compared to the control group, with soap bubble blowing being more effective in reducing fear (*p* < 0.001)	Level I	Strengths: RCT design, use of validated pain and fear scales; Weaknesses: Single setting, no long‐term follow‐up	High feasibility in clinical settings due to ease of implementation	Supports use of non‐pharmacological distraction methods like soap bubble blowing to manage pain and fear during pediatric procedures	Recommend using soap bubble blowing in pediatric settings to reduce procedural pain and fear
Semerci et al. [12]. The effect of buzzy and cold spray on pain, anxiety, and fear of children during venipuncture in pediatric emergency department in Turkey: A randomized controlled study	To evaluate the effectiveness of the Buzzy device and cold spray in reducing pain, anxiety, and fear in children during venipuncture	Gate Control Theory of Pain	Randomized Controlled Trial	161 children aged 5–12, pediatric emergency department, Turkey	Independent Variables: Buzzy device, cold spray; Dependent Variables: Pain, anxiety, and fear levels during venipuncture	Wong‐Baker FACES Pain Rating Scale, Child Anxiety Statement Scale, Child Fear Inventory	ANOVA and post hoc analysis	Both Buzzy and cold spray significantly reduced pain, anxiety, and fear compared to control; cold spray slightly more effective for pain	Level II	Strengths: Randomized control, direct measurement tools; Weaknesses: Single‐center, limited generalizability	High feasibility in clinical settings as both interventions are low‐cost and easy to implement	Buzzy and cold spray are effective non‐pharmacological options for managing procedural pain and anxiety in pediatric patients	Recommend routine use of Buzzy or cold spray in pediatric settings to reduce venipuncture distress
Sabetsarvestani et al. [16] A meta‐synthesis of the language of pediatric pain	To synthesize evidence on the language and communication strategies used to address pediatric pain	Communication Theory	Meta‐synthesis	Studies focusing on children aged 6–12 years, including various healthcare settings	Independent Variable: Language and communication strategies; Dependent Variable: Pain levels and coping mechanisms	Content analysis of qualitative data from included studies	Thematic Analysis	Effective communication strategies significantly improve pain perception and coping in pediatric populations	Level I	Strengths: Broad synthesis of multiple qualitative studies; Weaknesses: Limited focus on diverse cultural settings	Highly feasible as it involves training healthcare providers on effective communication	Effective communication is key to enhancing pain management and coping in pediatric patients	Recommend integrating communication training in healthcare provider education to improve pediatric pain management outcomes
Şahiner et al. [11] The effect of combined stimulation of external cold and vibration during immunization on pain and anxiety levels in children	To evaluate the efficacy of combined external cold and vibration in reducing pain and anxiety during immunization in children	Pain	Randomized Controlled Trial	120 children aged 6–12 years in a pediatric outpatient immunization clinic	Independent Variables: Combined external cold and vibration stimulation; Dependent Variables: Pain and anxiety levels during immunization	Wong‐Baker FACES Pain Rating Scale; Children's Anxiety Scale (self‐reported)	ANOVA	Combined external cold and vibration significantly reduced pain and anxiety levels compared to control	Level I	Strengths: Randomized design, objective and subjective measures of pain and anxiety; Weaknesses: Single‐center study, lack of long‐term follow‐up	Highly feasible for clinical pediatric settings; inexpensive and easy to administer	Combined cold and vibration stimulation is effective for pain and anxiety management during immunization	Recommend integrating this method as a standard practice in pediatric immunization settings to reduce procedural distress
Sarı et al. [21] Technology versus nostalgia: A randomized controlled trial of the effect of virtual reality and kaleidoscope on pediatric pain, fear, and anxiety management during immunization	To compare the efficacy of virtual reality (VR) and kaleidoscope use in managing pain, fear, and anxiety during pediatric immunizations	Pain	Randomized Controlled Trial	100 children aged 6–12 years in a pediatric outpatient immunization clinic	Independent Variables: VR and kaleidoscope; Dependent Variables: Pain, fear, and anxiety levels during immunization	Wong‐Baker FACES Pain Rating Scale, Children's Fear Scale (CFS), and self‐reported anxiety scales	ANOVA	VR significantly reduced pain and anxiety compared to kaleidoscope, which was more effective in fear reduction	Level I	Strengths: Randomized design, multi‐assessment of outcomes; Weaknesses: Single‐center study, relatively small sample size	Feasible for clinical settings with access to VR; kaleidoscopes are more practical in resource‐limited environments	VR and kaleidoscopes are effective tools for reducing procedural distress in children during immunization	Recommend using VR in technologically equipped facilities and kaleidoscopes as a cost‐effective alternative in resource‐limited settings
Suleiman‐Martos et al. [27] Effect of a game‐based intervention on preoperative pain and anxiety in children: A systematic review and meta‐analysis	To evaluate the effectiveness of game‐based interventions on preoperative pain and anxiety in pediatric populations	Pain	Systematic review and meta‐analysis	Data from 15 studies with a total of 1200 pediatric participants undergoing preoperative procedures in various clinical settings	Independent Variables: Game‐based interventions (e.g., video games, VR); Dependent Variables: Pain and anxiety levels	Wong‐Baker FACES Pain Rating Scale and self‐reported anxiety measures	Meta‐analysis	Game‐based interventions significantly reduced preoperative pain and anxiety levels compared to standard care	Level I	Strength: Inclusion of diverse study designs; Weakness: Variation in intervention implementation across studies	Highly feasible for integration in clinical settings with minimal resource constraints	Game‐based interventions are effective for preoperative pain and anxiety management in children	Recommend integrating game‐based interventions into standard preoperative care for children
Wong and Choi [17] Effects of an immersive virtual reality intervention on pain and anxiety among pediatric patients undergoing venipuncture: A randomized clinical trial	To examine the effects of immersive VR interventions on reducing pain and anxiety in pediatric patients undergoing venipuncture	Cognitive‐Behavioral Framework	Randomized Controlled Trial	150 children aged 6–12 years from a pediatric outpatient clinic	Independent Variable: Immersive VR intervention; Dependent Variables: Pain and anxiety levels during venipuncture	Wong‐Baker FACES Pain Rating Scale and self‐reported anxiety scales	RM‐ANOVA	Immersive VR significantly reduced pain and anxiety levels compared to standard care	Level I	Strength: Strong randomization and use of validated measures; Weakness: Single clinical setting may limit generalizability	High feasibility for clinics with access to VR equipment and trained staff	Immersive VR is effective for reducing procedural pain and anxiety in pediatric patients	Recommend integrating VR interventions in outpatient pediatric care settings to manage procedural distress
Yılmaz et al. [24] Efficacy of Buzzy on pain and anxiety during catheterization in children	To assess the efficacy of the Buzzy device in reducing pain and anxiety in children undergoing peripheral intravenous catheterization	Gate Control Theory of Pain	Randomized Controlled Trial	60 children aged 8–16, conducted in a children's hospital in Turkey	Independent Variable: Use of Buzzy device; Dependent Variables: Pain and anxiety levels during catheterization	Wong‐Baker Faces Pain Rating Scale, Children's Fear Scale (CFS)	Mann–Whitney U test	No significant difference found between the pain and anxiety levels of the Buzzy group and control group; Buzzy did not reduce pain and anxiety effectively in this procedure	Level II	Strengths: Randomized controlled design; Weaknesses: Small sample size, limited to one hospital, non‐blinded	Feasibility in clinical settings, though Buzzy may be less effective for certain invasive procedures in older children	The study concluded that Buzzy was not effective in reducing pain and anxiety in peripheral intravenous catheterization for this age group	Further research recommended on the effectiveness of Buzzy in other settings and for different procedures

*Note:* Levels of evidence are classified according to standard hierarchies used in nursing research, with Level I indicating the highest level of evidence (systematic reviews, meta‐analyses, or randomized controlled trials) and lower levels indicating progressively weaker designs.

Abbreviations: CFS, Children's Fear Scale; LOE, level of evidence; RCT, randomized controlled trial; VAS, Visual Analog Scale; VR, virtual reality.

**TABLE A3:** Level of evidence synthesis: Summary of interventions across included studies.

Studies	Intervention			
	AI‐based interactive games	Buzzle bee	VR*	Other distraction methods
Alemdar and Aktaş [5]		x		
Arıkan and Işık Esenay [26]				x
Binay et al. [13]		x		
Cheraghi et al. [14]				x
Cozzi et al. [15]				x
Dumoulin et al. [18]			x	
Eijlers et al. [19]			x	
Erdogan and Aytekin Ozdemir [31]		x	x	
Gerçeker et al. [23]			x	
Girgin and Göl [6]				x
Guillari et al. [28]		x	x	
Johnson [20]			x	
Kara et al. [29]				x
Karakaya and Gözen [25]				x
Le May et al. [32]			x	
Lluesma‐Vidal et al. [9]			x	
Ahmed et al. [4]		x		
Merino‐Lobato et al. [33]		x	x	
Moadad et al. [34]		x		
Oluc and Arslan [7]				x
Sabetsarvestani et al. [16]				x
Şahiner et al. [11]			x	
Sarı et al. [21]			x	
Semerci et al. [12]		x		
Suleiman‐Martos et al. [27]				x
Tas et al. [10]			x	
Wong and Choi [17]			x	
Yaz et al. [22]		x		
Yılmaz et al. [24]		x		

*Note:* AI‐based interactive game studies were limited in the current nursing and pediatric pain literature. VR studies were therefore included as related technology‐based distraction evidence, but VR was not treated as equivalent to AI‐based interactive games. A check mark (x) indicates that the study evaluated the corresponding intervention.

*VR: Virtual Reality.

**TABLE A4:** Detailed evidence summary for included studies.

Citation: Author, date of publication and title	Design/Method	Sample/Setting	Findings	Outcome
Ahmed et al. [4] Effect of Buzzy and watching cartoons on venipuncture pain among children undergoing phlebotomy.	Randomized Controlled Trial	Pediatric patients aged 4–12 years undergoing venipuncture in an outpatient pediatric clinic in Egypt	Significant reduction in pain and anxiety for both interventions, with the combined use of Buzzy and cartoons being the most effective	Supports the combined use of Buzzy and visual distractions (cartoons) for reducing pain and anxiety during pediatric venipuncture
Alemdar and Aktaş [5]	Randomized Control Trial (RCT)	Pediatric patients aged 5 to 10 years undergoing venipuncture in a maternity and children's hospital	Significant effect of Buzzy and bubble‐blowing on reducing pain and fear during venipuncture	Supports using non‐pharmacological interventions during venipuncture
Binay et al. [13] Comparison of the effectiveness of two different methods of decreasing pain during phlebotomy in children: A randomized controlled trial	Randomized Controlled Trial	96 children aged 3–6 years, university hospital pediatric phlebotomy department in Turkey	Pain scores were significantly lower in both intervention groups compared to the control group; no significant difference between the two intervention methods	Both external cold and vibration and blowing soap bubbles are effective non‐pharmacological methods for reducing procedural pain in children
Carr et al. [1]	Cohort Study	Pediatric patients, ages 3–12, in hospital settings	Older (aged 8–12) children reported less pain and fear, while gender differences were not consistently significant, except that girls reported more pain than boys	Age is a factor in pain and fear, while gender differences are less consistent
Cheraghi et al. [14] A Comparison of the Effect of Visual and Auditory Distractions on Physiological Indicators and Pain of Burn Dressing Change Among 6–12‐Year‐Old Children: A Clinical Trial Study	Single‐blind clinical trial	120 children (6–12 years) in a burn unit, Iran	Both distractions significantly reduced pain, with visual distraction showing stronger effects on pain reduction and physiological stability	Visual and auditory distraction are effective for reducing pain and improving physiological stability during painful procedures in pediatric burn care
Cohen et al. [2]	Systematic Review (examining 17 different pain assessment tools)	Various settings, focusing on pediatric pain assessments (reviews a broad range of settings and populations related to pediatric pain, including infants, children, and adolescents, with various pain measures.)	The effectiveness of various pain assessment tools and mention cognitive‐behavioral techniques as useful strategies	Cognitive‐behavioral techniques are recommended for procedural pain
Cozzi et al. [15] A narrative review with practical advice on how to decrease pain and distress during venepuncture and peripheral intravenous cannulation	Narrative Review	Studies involving pediatric venipuncture and intravenous cannulation	Highlighted the effectiveness of distraction, physical comfort, and parental involvement in reducing procedural pain and distress	Emphasizes the importance of preparation, parental support, and distraction techniques in pediatric procedural pain management
Dumoulin et al. [18] A randomized controlled trial of the use of virtual reality for needle‐related procedures in children and adolescents in the emergency department	Randomized Controlled Trial	94 children and adolescents (ages 6–17 years) undergoing needle‐related procedures in an emergency department	VR significantly reduced pain and distress during needle‐related procedures compared to standard care	Supports VR as an effective distraction tool for managing needle‐related procedural pain and distress in pediatric emergency settings
Eijlers et al. [19] Meta‐analysis of Virtual Reality in Pediatrics: Effects on Pain and Anxiety	Systematic Review and Meta‐Analysis	Pediatric patients across multiple studies and various settings	VR significantly reduced both pain (SMD = −1.30) and anxiety (SMD = −1.32) in pediatric patients during procedures	VR is an effective non‐pharmacological tool for reducing pain and anxiety in pediatric procedures
Erdogan and Aytekin Ozdemir [31]. The Effect of Three Different Methods on Venipuncture Pain and Anxiety in Children: Distraction Cards, Virtual Reality, and Buzzy	Randomized Controlled Trial	142 children aged 7–12 years undergoing venipuncture in a university hospital	Buzzy group reported the lowest pain and anxiety scores, followed by VR and Distraction Cards groups, indicating effectiveness in pain and anxiety reduction	Non‐pharmacological interventions like Buzzy, VR, and Distraction Cards can be effective in reducing pain and anxiety during pediatric procedures
Gerçeker et al. [23] The effects of virtual reality and stress ball distraction on procedure‐related emotional appearance, pain, fear, and anxiety during phlebotomy in children: A randomized controlled study	Randomized Controlled	150 children aged 7–12 years in a pediatric phlebotomy clinic in Turkey	VR significantly reduced pain, fear, and anxiety compared to both the control and stress ball groups. VR also reduced negative emotional behaviors post‐procedure. Le	VR is effective in reducing pain, fear, anxiety, and negative emotional behaviors in children, showing potential for clinical application in pediatric phlebotomy
Girgin and Göl [6]	Randomized Control Trial (RCT)	Pediatric patients, ages 7–12, undergoing venipuncture	Distraction techniques (balloon inflation, ball squeezing, and coughing) significantly reduced both pain and fear during venipuncture compared to the control group	Distraction should be incorporated as a standard intervention as they are effective in reducing pain and fear
Guillari et al. [28] Non‐pharmacological interventions to reduce procedural needle pain in children (6–12 years):	Systematic Review	15 studies including 1356 pediatric patients across various healthcare settings	VR and cold‐vibration devices were the most effective interventions for reducing procedural pain and anxiety, particularly in school‐aged children	Non‐pharmacological interventions, particularly VR and cold‐vibration devices, are effective tools for reducing pain and anxiety in pediatric populations
Johnson [20] Virtual reality for pain management in children	Narrative Review	Not applicable	VR is a promising tool for reducing pain and anxiety in children, with significant potential for integration into various pediatric healthcare settings	Supports the adoption of VR as a non‐pharmacological intervention to manage pain and anxiety in pediatric patients
Kara et al. [29] Interactive Games and Pediatric Pain Management.	Randomized Controlled Trial	120 pediatric patients aged 6–12 years undergoing venipuncture in a hospital setting	Interactive games significantly reduced pain and anxiety compared to standard care, with greater engagement observed among younger children	Supports the integration of interactive games into procedural pain management for pediatric patients
Karakaya and Gözen [25] The effect of distraction on pain level felt by school‐age children during venipuncture procedure: A randomized controlled trial	Randomized Controlled Trial	80 school‐aged children (6–12 years old) in a hospital outpatient setting	Distraction techniques significantly reduced pain levels compared to the control group, with higher efficacy in younger children	Distraction techniques are effective and should be included as a standard practice in pediatric venipuncture procedures
Le May et al. [32] Decreasing pain and fear in medical procedures with a pediatric population	Pilot Randomized Within‐Subject Trial	30 pediatric patients aged 6–12 years in a hospital setting	VR significantly reduced pain and fear compared to the control group receiving standard care	VR is an effective tool for reducing pain and fear in pediatric procedures and should be considered in clinical settings
Lluesma‐Vidal et al. [9] Effect of Virtual Reality on Pediatric Pain and Fear During Procedures Involving Needles	Systematic Review and Meta‐Analysis	21 studies reviewed, including 10 for meta‐analysis with a total sample of pediatric patients across various healthcare settings	VR was effective in significantly reducing pain (mean reduction of 2.37) and fear (mean reduction of 1.26) in children undergoing needle procedures	VR is an effective non‐pharmacological tool for reducing pain and fear in pediatric procedures involving needles
Ahmed et al. [4]	Quasi‐experimental	The sample consisted of 150 school‐age children undergoing phlebotomy at pediatric emergency units	Both Buzzy and cartoons significantly reduced pain, with Buzzy being more effective	Both Buzzy and cartoons were effective in reducing pain during venipuncture
[33] Virtual reality vs. Buzzy: Efficacy in pain and anxiety management during pediatric venipuncture	Systematic Review & Meta‐Analysis	21 studies with a total sample of 2504 pediatric patients (age 2–18) from various healthcare settings	Both VR and Buzzy significantly reduced pain and anxiety compared to standard care; no significant difference in efficacy between VR and Buzzy	Supports VR and Buzzy as effective non‐pharmacological interventions for managing pain and anxiety during venipuncture in children
Moadad et al. [34] Distraction Using the BUZZY for Children During an IV Insertion	Randomized Controlled Trial	48 children aged 4–12 years, hospital setting	BUZZY group showed significantly lower pain scores compared to control, indicating effective pain reduction	BUZZY is effective for reducing pain perception during IV insertions in children
Oluc and Arslan [7]	Randomized Control Trial (RCT)	The sample consisted of 111 children aged 6–12 years undergoing phlebotomy in an emergency department	Both soap bubble blowing, and ball squeezing significantly reduced pain and fear compared to the control group. However, soap bubble blowing was more effective in reducing fear than ball squeezing	Both methods are viable in clinical practice. The method of soap bubble blowing was found to be a more effective method in reducing fear
Oluc and Arslan [7] The effect of two different methods on reducing the pain and fear during phlebotomy to children: A randomized controlled trial	Randomized Controlled	111 children aged 6–12 years in an emergency department in Turkey	Both soap bubble blowing and ball squeezing significantly reduced pain and fear compared to the control group, with soap bubble blowing being more effective in reducing fear (*p* < 0.001)	Supports use of non‐pharmacological distraction methods like soap bubble blowing to manage pain and fear during pediatric procedures
Sabetsarvestani et al. [16] A meta‐synthesis of the language of pediatric pain	Meta‐synthesis	Studies focusing on children aged 6–12 years, including various healthcare settings	Effective communication strategies significantly improve pain perception and coping in pediatric populations	Effective communication is key to enhancing pain management and coping in pediatric patients
Şahiner et al. [11] The effect of combined stimulation of external cold and vibration during immunization on pain and anxiety levels in children	Randomized Controlled Trial	120 children aged 6–12 years in a pediatric outpatient immunization clinic	Combined external cold and vibration significantly reduced pain and anxiety levels compared to control	Combined cold and vibration stimulation is effective for pain and anxiety management during immunization
Sarı et al. [21] Technology versus nostalgia: A randomized controlled trial of the effect of virtual reality and kaleidoscope on pediatric pain, fear, and anxiety management during immunization	Randomized Controlled Trial	100 children aged 6–12 years in a pediatric outpatient immunization clinic	VR significantly reduced pain and anxiety compared to kaleidoscope, which was more effective in fear reduction	VR and kaleidoscopes are effective tools for reducing procedural distress in children during immunization
Schechter et al. [3]	Systematic Review	Pediatric patients during immunizations	Behavioral interventions reduce pain	Behavioral strategies, such as distraction and parental demeanor, are effective in reducing pain during pediatric immunizations
Semerci et al. [12]. The effect of buzzy and cold spray on pain, anxiety, and fear of children during venipuncture in pediatric emergency department in Turkey: A randomized controlled study	Randomized Controlled Trial	161 children aged 5–12, pediatric emergency department, Turkey	Both Buzzy and cold spray significantly reduced pain, anxiety, and fear compared to control; cold spray slightly more effective for pain	Buzzy and cold spray are effective non‐pharmacological options for managing procedural pain and anxiety in pediatric patients
Suleiman‐Martos et al. [27] Effect of a game‐based intervention on preoperative pain and anxiety in children: A systematic review and meta‐analysis	Systematic review and meta‐analysis	Data from 15 studies with a total of 1200 pediatric participants undergoing preoperative procedures in various clinical settings	Game‐based interventions significantly reduced preoperative pain and anxiety levels compared to standard care	Game‐based interventions are effective for preoperative pain and anxiety management in children
Tas et al. [10]	Systematic Review and Meta‐Analysis	The study includes pediatric patients (children and adolescents) undergoing various medical procedures. It extended the earlier review's search to 2020, leading to the inclusion of 26 articles	Virtual reality significantly reduces pain and anxiety	VR is an effective tool for managing pain and anxiety in pediatric patients during medical procedures
Wong and Choi [17] Effects of an immersive virtual reality intervention on pain and anxiety among pediatric patients undergoing venipuncture: A randomized clinical trial	Randomized Controlled Trial	150 children aged 6–12 years from a pediatric outpatient clinic	Immersive VR significantly reduced pain and anxiety levels compared to standard care	Immersive VR is effective for reducing procedural pain and anxiety in pediatric patients
Yaz et al. [22]	Randomized Control Trial (RCT)	The sample was 105 children aged 3–6 undergoing phlebotomy in a pediatric unit	The study found that both Bee Buzzy and puppets were effective in reducing pain and fear, although Bee Buzzy was more effective for pain	The article concludes that both methods were effective, with Bee Buzzy more effective for pain reduction
Yılmaz et al. [24] Efficacy of Buzzy on pain and anxiety during catheterization in children	Randomized Controlled Trial	60 children aged 8–16, conducted in a children's hospital in Turkey	No significant difference found between the pain and anxiety levels of the Buzzy group and control group; Buzzy did not reduce pain and anxiety effectively in this procedure	The study concluded that Buzzy was not effective in reducing pain and anxiety in peripheral intravenous catheterization for this age group
Yılmaz et al. [8]	Randomized Control Trial (RCT)	The study included 90 healthy adult males voluntarily donating blood at a university blood center	The study found a statistically significant reduction in pain and an increase in satisfaction in the Buzzy group compared to the control groups	Buzzy is effective in reducing pain and increases satisfaction during phlebotomy
Young [30]	Review Article	The article addresses pain in pediatric settings, focusing on routine and emergency medical procedures, such as immunizations and venipunctures	The article provides a detailed review of the current understanding of pediatric pain and offers evidence‐based recommendations for management	Pain management should be a priority for pediatric care

*Note:* This table presents the design, sample characteristics, setting, primary findings, and outcomes reported in each included study.
